# Significance of Preoperative Systemic Immune Score for Stage I Gastric Cancer Patients

**DOI:** 10.1155/2018/3249436

**Published:** 2018-07-11

**Authors:** Jun Lu, Long-long Cao, Ping Li, Jian-wei Xie, Jia-bin Wang, Jian-xian Lin, Qi-yue Chen, Mi Lin, Ru-hong Tu, Chang-ming Huang, Chao-hui Zheng

**Affiliations:** ^1^Department of Gastric Surgery, Fujian Medical University Union Hospital, Fuzhou, China; ^2^Department of General Surgery, Fujian Medical University Union Hospital, Fuzhou, China; ^3^Key Laboratory of Ministry of Education of Gastrointestinal Cancer, Fujian Medical University, Fuzhou, China; ^4^Fujian Key Laboratory of Tumor Microbiology, Fujian Medical University, Fuzhou, China

## Abstract

**Background:**

Determining preferences regarding the benefits of adjuvant chemotherapy (AC) for stage I GC is critical.

**Methods:**

We retrospectively reviewed 1069 patients with pathologically confirmed stage I GC who underwent R0 gastrectomy between 2006 and 2014. Univariate and multivariate survival analyses were conducted. Systemic inflammation factors were used to develop a scoring system for predicting AC benefits.

**Results:**

With a median follow-up of 47 months (range 3–113 months), the 5-year overall survival (OS) rate was 90.5%. The patient score was 1 for either a pretreatment hypoalbuminemia or elevated derived neutrophil-lymphocyte ratio (dNLR) and was 0 otherwise. The SIS served as an independent prognostic factor for reduced OS. AC was delivered to 13.5% (144/1069) of all patients. Compared to surgery alone, AC had no significant effect on survival in both the entire cohort and the IA/IB subgroup. However, in the high-risk group (SIS = 2), patients with AC had a significantly better OS than those undergoing surgery alone.

**Conclusions:**

Patients with SIS = 2 may benefit from AC and thus may be considered candidates for adjuvant treatment. However, to confirm our findings, future prospective studies are warranted.

## 1. Introduction

Gastric cancer (GC) is the fourth most common cancer and the second leading cause of cancer death worldwide [1]. Unlike the situation for stage II and stage III GC, there is no global agreement on adjuvant chemotherapy (AC) for stage I GC due to (1) the prognosis of stage I GC is relatively good, with over 90% of 5-year overall survival [2], (2) although the benefit of AC was established for patients with stage II and stage III GC, clinical trials included stage I population failed to show the benefit of AC over surgery alone [2–7]. However, relapse occurs in a small but definite number of patients, even those with stage I GC, after curative resection [8]. Moreover, recent studies showed that the prognosis and response to chemotherapy is different according to the molecular characteristics of gastric cancer [9–11]. Therefore, it is necessary to identify patients with stage I GC who might receive a therapeutic benefit from AC.

The role of the immune system in cancer has become increasingly prominent [12–17]. In addition to local inflammatory symptoms, cancer patients frequently present with systemic inflammation responses, including increased peripheral blood cell amounts and decreased serum albumin levels [18]. More recently, a study at the Memorial Sloan Kettering Cancer Center suggested that pretreatment neutrophil lymphocyte ratio (NLR) can be used to identify nonmetastatic melanoma patients who are more likely to benefit from adjuvant treatment [19]. In 2012, Proctor et al. [20] implemented a simplified index that is easier to apply to clinical data named the derived neutrophil-lymphocyte ratio (dNLR), and they were also able to demonstrate that the preoperative dNLR had similar prognostic value as the classical NLR. Preoperative serum albumin levels are also reported as prognostic indicators for the prognosis of cancer patients [21, 22]. These markers are inexpensive to test and routinely performed in clinical setting and hence potentially provide readily available and objective information to help clinicians to estimate patient outcome.

To the best of our knowledge, the potential influence of the pretreatment dNLR and hypoalbuminemia has never been explored in large cohorts of stage I GC patient yet. The aim of the present study was to verify the hypothesis that the systemic immune score (SIS) based on preoperative dNLR and serum albumin is associated with worse survival in diagnosed stage I GC and more importantly to investigate whether SIS can distinguish subgroups of patients who would benefit from AC. If so, such commonly measured SIS could be used in developing a strategy for selecting stage I GC treatments.

## 2. Materials and Methods

### 2.1. Patients

A total of 1069 patients undergoing R0 resection for stage I GC at Fujian Medical University Union Hospital (FMUUH) from December 2006 to December 2016 were identified from a prospectively maintained database. The following exclusion criteria were then applied: (1) prior gastrectomy, (2) receiving neoadjuvant chemotherapy, (3) noncurative (R1/2) resection, (4) the presence of synchronous malignant disease, (5) autoimmune disorders and recent steroid therapy, and (6) incomplete medical records. Curative resection refers to R0 resection in Japanese Gastric Cancer Treatment Guidelines 2010 (version 3) [23]. Tumor stages were assessed according to the American Joint Committee on Cancer (AJCC) classification system, 7th edition [24]. Additional demographic and clinical data are summarized in Table 1. The study protocol was approved by the ethical committee of the FMUUH.

### 2.2. Adjuvant Chemotherapy

Since there is no established treatment strategy for stage I gastric cancer, decisions to administer AC in those patients were based on their surgeons' or oncologists' preference [25, 26]. According to the treatment protocol of our institute, stage I GC patients with lymph node metastasis, lymphovascular invasion, or other high risks [27, 28] are recommended for AC, unless contraindicated by a patient's condition or their refusal. All AC treatments were performed after individually obtaining written informed consent in accordance with the gastric cancer treatment program at the FMUUH that had been approved by its institutional review board. Finally, 144 (13.5%) patients with written informed consent received 5-fluorouracil plus cisplatin AC, at least one cycle [29]. The clinicopathological variables of both groups are summarized in Supplementary Table 1.

### 2.3. Systemic Immune Markers

Routine clinical laboratory analyses of peripheral blood samples collected within 1 week before the operation were used to ascertain white cell counts, neutrophil counts, and serum albumin. The pretreatment dNLR was calculated as follows: dNLR = neutrophil count to (white cell count-neutrophil count). The cutoff value (2.0) for the pretreatment dNLR was selected as described previously [20]. The cutoff value for the pretreatment hypoalbuminemia (35 g/l) was according to the normal value measured by the used test [22, 30]. All the measurements were performed in the central biochemical laboratory at the Department of Clinical Biochemistry and Hematology, FMUUH.

We combined the two risks to establish the SIS defined as follows: patients with both elevated dNLR and hypoalbuminemia (dNLR ≥ 2 and albumin < 35 g/l, resp.) were assigned score 2, patients with either elevated dNLR or hypoalbuminemia were assigned score 1, and patients with both decreased dNLR and normal albumin (dNLR < 2 and albumin ≥ 35 g/l, resp.) were assigned score 0.

### 2.4. Follow-up

All patients were monitored postoperatively by physical examination and laboratory tests, including those for tumor markers (such as CEA and CA 19–9), every 3 months for the first 2 years, every 6 months for the next 3 years, and annually thereafter. In addition, examinations, including chest radiography, abdominopelvic computed tomography (CT), and endoscopy, were performed at least once a year. If necessary, further evaluation, such as positron emission tomography or magnetic resonance imaging, was initiated.

### 2.5. Statistical Analysis

Overall survival (OS) was defined as the time from the day of surgery to the death from any cause. Univariate survival trends were compared using Kaplan-Meier curves and significant differences determined via the log rank test, and those variables that achieved statistical significance in the univariate analysis were entered into the multivariable analysis. Multivariate analysis of prognostic factors was conducted with Cox's proportional hazards model. A *p* value < 0.05 was regarded statistically significant. Statistical analysis was performed using SPSS version 19.0 for Windows (SPSS Inc., Chicago, IL, USA).

## 3. Results

### 3.1. Patient Characteristics

A total of 1069 patients with pathologically documented stage I GC were included in this study. The study population comprised of 795 (74.4%) males and 274 (25.6%) females. The median age was 60 years (range 18–93). Descriptive clinicopathological and detailed blood count parameters of the study cohort are shown in Table 1. The percentage of patients with dNLR ≥ 2 was 25.4% (272/1069) in contrast to 74.6% (797/1069) for those with dNLR < 2. Pretreatment hypoalbuminemia (albumin < 35 g/l) occurred in 174 patients (16.3%). Overall, 925 (86.5%) patients received surgery alone (the non-AC group), and 144 (13.5%) received AC after surgery (the AC group). The baseline characteristics between the groups are shown in Table 2.

### 3.2. Survival

The 5-year OS rates in T1N0, T1N1, and T2N0 were 95.7%, 85.3%, and 80.0%, respectively (*p* < 0.001). There were significant differences in 5-year OS between patients with T1N0 and T1N1 (*p* < 0.001) or T2N0 tumors (*p* < 0.001). However, no difference was observed in OS between patients with T1N1 tumors and T2N0 tumors (*p* = 0.461) (Supplementary Figure 1a). The 5-year OS was significantly higher in stage IA patients than in stage IB patients (Supplementary Figure 1b).

**Figure 1:**
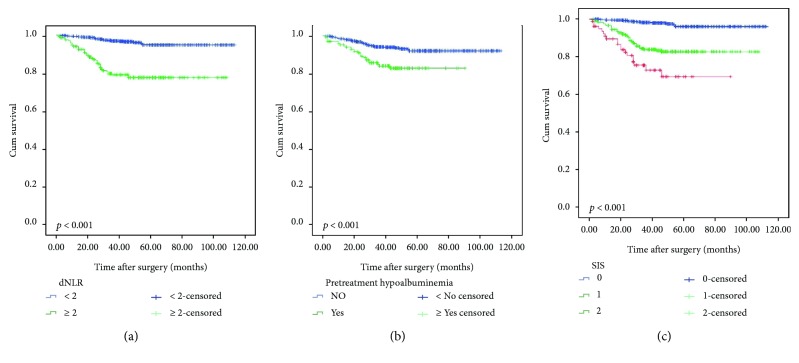
Kaplan-Meier curves for high versus low dNLR (a), with versus without pretreatment hypoalbuminemia (b), and patients according to different SIS (c) in the entire cohort.

The 5-year OS was significantly worse in high dNLR patients than in low dNLR patients (77.6% versus 94.7%, *p* < 0.001; Figure 1(a)). Patients with and without pretreatment hypoalbuminemia also differed significantly in 5-year OS (82.9% versus 92.1%, *p* < 0.001; Figure 1(b)).

### 3.3. Predictive Factors for Survival

The statistically significant prognostic factors identified by univariate analyses are shown in Table 3. Based on the multivariate analysis, the elevated pretreatment dNLR and hypoalbuminemia were independent prognostic factors for OS, together with TNM stage and lymphovascular invasion (Table 3).

### 3.4. Establishment and Prognostic Impact of the Novel Systemic Immune Score (SIS)

Based on the systemic immune prognostic factors identified in the multivariate analysis, we combined the two factors to establish the SIS defined as follows: patients with both high dNLR and the pretreatment hypoalbuminemia were assigned a score of 2, patients with either high dNLR or the pretreatment hypoalbuminemia were assigned a score of 1, and patients with both low dNLR and normal albumin were assigned a score of 0. Namely, patients were given a total score of 0, 1, and 2 based on the sum of the points.

As mentioned above, we generate three subgroups based on the SIS. We found significant differences among the three subgroups. Thus, we combined the three subgroups to establish the SIS classification as follows: the low-risk group has no risk factor (SIS = 0), the medium-risk group has 1 risk factor (SIS = 1), and the high-risk group has two risk factors (SIS = 2) (Figure 1(c)). The SIS classification also was an independent prognostic factor for OS (Table 3). Supplementary Figure 2 depicts the association between the SIS and TNM as a bubble chart.

### 3.5. SIS Predicts the Benefits of Adjuvant Chemotherapy

**Figure 2:**
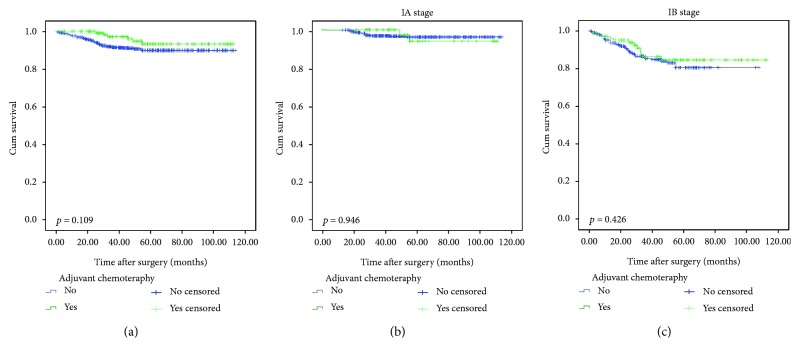
Comparison of OS between the adjuvant chemotherapy group and the surgery-only group. (a) Entire group, (b) IA stage subgroup, and (c) IB stage subgroup.

**Figure 3:**
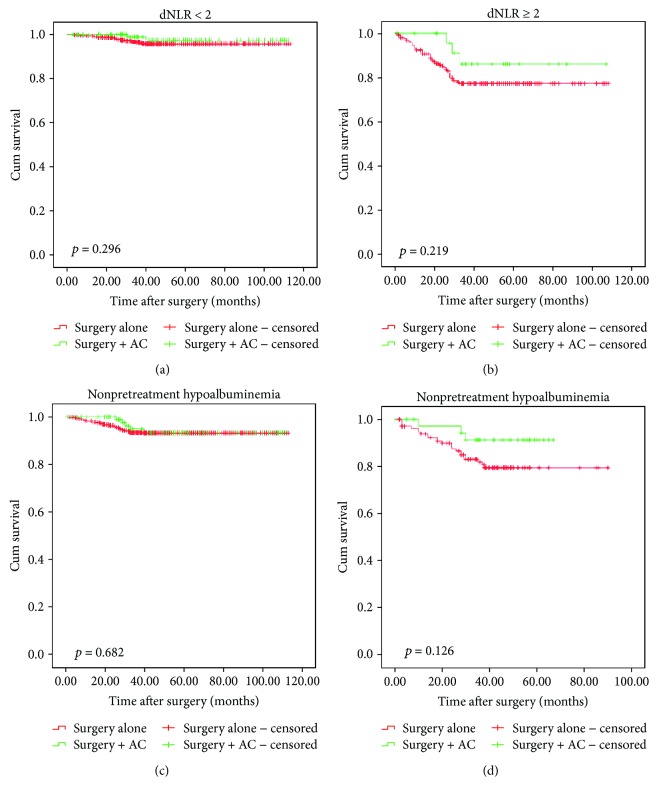
Comparison of OS between adjuvant chemotherapy group and surgery-only group. (a) dNLR < 2 subgroup, (b) dNLR ≥ 2 subgroup, (c) hypoalbuminemia subgroup, and (d) nonhypoalbuminemia subgroup.

Figure 2(a) shows the survival curves for patients with and without AC. In the entire cohort, there was no significant difference in 5-year OS between the groups with and without AC (93·4% versus 90.0%, *p* = 0.109). This lack of a difference was also apparent when the analysis was confined to stage IA (93.7 versus 96.0%, *p* = 0.946) and IB (83.7% versus 79.5%, *p* = 0.426) subgroup patients (Figures 2(b) and 2(c)). Moreover, the survival curves (Figure 3) show no difference between surgery alone versus surgery + AC according to pretreatment dNLR and hypoalbuminemia.

**Figure 4:**
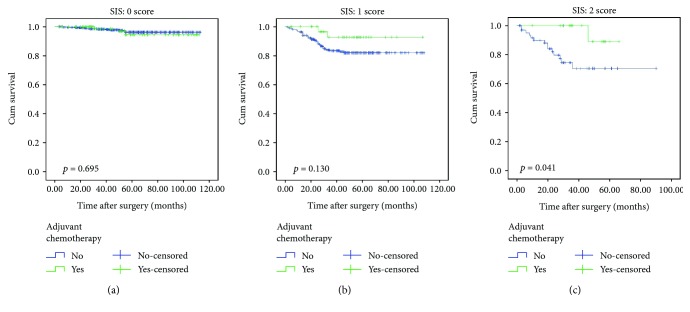
Comparison of OS between adjuvant chemotherapy group and surgery-only group. (a) 0 score subgroup, (b) 1 score subgroup, and (c) 2 score subgroup.

Surprisingly, for our original SIS system, Kaplan-Meier curves demonstrated that high-risk patients (SIS = 2) with AC had significantly better OS than their counterparts without AC (88.9% versus 69.4%, *p* = 0.023) (Figure 4(a)), while no significant difference was observed in the low-risk and medium-risk group (94.4% versus 97.6%, *p* = 0.695 and 91.6% versus 81.9%, *p* = 0.130) (Figures 4(b) and 4(c)). In other words, the survival benefit of AC was significant for patients with SIS = 2.

## 4. Discussion

With the increase in the detection of stage I GC, the number of stage I GC patients who experience recurrence has also increased, which may result in a large health problem in the real world [2, 8]. It is thus important to identify patients with apparent early-stage disease who will not be cured by surgery alone and should receive adjuvant chemotherapy (AC). To date, almost all of the data on AC are derived from large-scale clinical trials that included a large proportion of stages II and III patients but no or too few patients with stage I GC [3–6]. A prospective randomized clinical trial of AC versus surgery alone in stage I GC patients at high risk of recurrence or death is still ongoing (ClinicalTrials.gov identifier NCT01917552). Therefore, it will be clinically useful if a score system can be used to select candidates for AC.

A systemic inflammatory response has been determined to be an important tumor stage-independent predictor of prognosis in various malignances [31]. Pretreatment NLR values are associated with prognosis in various solid tumors, including gastric adenocarcinoma [15, 17, 32]. In 2012, Proctor et al. [20] reported the dNLR and NLR have similar prognostic value in a large cohort of unselected cancer patients. More recently, Dalpiaz [33] and his colleges found that the pretreatment dNLR was better than NLR in terms of acting as an independent prognostic factor for cancer patients. In this study, we also clearly demonstrate that dNLR was an independent predictor of long-term survival for stage I GC patients (*p* < 0.001).

In addition to pretreatment NLR, another systemic immune factor was predictive of prognosis in stage I GC in our cohort: pretreatment hypoalbuminemia. There is a growing appreciation that pretreatment hypoalbuminemia can influence the prognosis of cancer patients, possibly through a systemic inflammatory response or impaired immunological response [34]. A recent meta-analysis of 29 studies on variety of gastrointestinal tract solid tumors reported that pretreatment serum albumin levels provide useful prognostic significance in cancer [30]. Several studies have found that low serum albumin level was an independent prognostic factor for worse outcomes in patients with gastric cancer [35, 36]. In line with previous studies, the results of the current study show an association between pretreatment hypoalbuminemia and decreased 5-year OS.

As stated above, dNLR and hypoalbuminemia were independent prognostic predictors of survival in patients with stage I GC. Consequently, we created a novel prognostic score named the SIS based on the combination of dNLR and hypoalbuminemia, and we hypothesize that the SIS may provide further information in addition to that from classical pathological prognostic factors for identifying patients who are at high risk for poor prognosis. By grouping the patients on the basis of SIS, we identified three risk groups with distinct survival outcomes, that is, a high-risk group (SIS = 2) with a 5-year OS rate of 71.2%, a medium-risk group (SIS = 1) with a 5-year OS rate of 83.1%, and a low-risk group (SIS = 0) with a 5-year OS rate of 96.1%. When the SIS is used for risk stratification, stage I GC patients could be categorized into three different risk groups displaying a 5-year OS rate difference of almost 25 percentage points.

Proceeding to the next step, NLR can be used to assist in risk stratification and potentially predicting immunotherapy treatment response of patients with nonmetastatic melanoma [19]. Another study from France found that pretreatment serum albumin level > 35 g/l was the only independent predictive factor of complete response to chemoradiotherapy (CRT) in esophageal cancer patients [37]. Therefore, we hypothesized that SIS may be used to detect patients who can benefit from AC in stage I GC. For stage I gastric cancer, poorly differentiated or higher-grade cancer, LBVI, PNI, or <50 years of age are considered high-risk features, and adjuvant therapy is recommended in these high-risk patients [27]. Kunisaki et al. [38] revealed that depth of invasion, lymph node metastasis, and LBVI independently influenced prognosis in stage I gastric cancer, and in their report, patients with stage I GC having lymphovascular invasion and stage II GC had similar survival outcomes; this finding suggests that adjuvant chemotherapy is suitable for stage I patients with moderate to severe lymphovascular invasion. However, none of the previous studies compared the prognosis between patients with AC and those without AC. Notably, distinct from the previous studies, we first directly compared the survival of stage I GC patients with or without AC in terms of differences in inflammation-immune status. The patients with an SIS = 2 could achieve significantly better prognosis if treated with AC and should thus be considered candidates for adjuvant treatment.

There were some limitations of the present study. First, it is a single-center retrospective study. Second, we did not perform external or internal validation of our risk model. However, it might be difficult to construct a dataset including a patient population as large as ours for external validation. Despite the large cohort size in this study, the number of events was considered not insufficient to divide patients into training and validation sets for internal validation, and the number of high-risk patients was relatively small. The prognostic significance of the SIS in GC patients remains to be investigated prospectively in other populations and larger cohorts in the future. Third, we did not compare the prognostic value of other SIS, such as PNI, PLR, NLR, and GPS. Finally, the optimal AC cycle for stage I patients had not yet been established.

## 5. Conclusions

The present study demonstrated that stage I GC patients with elevated pretreatment dNLR and pretreatment hypoalbuminemia were likely to receive a survival benefit from AC. In view of this, the SIS should be included in routine clinical assessments and might also be considered for risk stratification in future clinical trials of AC in early-stage GC. However, it is still necessary to conduct prospective clinical validation studies to confirm the findings of this study.

## Figures and Tables

**Table 1:** Demographic and clinical features.

Characteristics	Number	%	Characteristics	Number	%
Age, years (median, IQR)	60 (18–93)		ASA class		
Sex			1-2	1020	95.4
Male	795	74.4	≥3	49	4.6
Female	274	25.6	Type of surgery		
CEA level			Total gastrectomy	284	26.6
Normal	871	81.5	Subtotal gastrectomy	785	73.4
Above normal	116	10.9	Type of LN dissection		
Unknown	82	7.7	D1	11	1.0
CA 19–9 level			D1+	549	51.3
Normal	826	77.3	D2	509	47.7
Above normal	152	14.2	Lymphovascular invasion		
Unknown	91	8.5	No	945	88.4
Differentiation			Yes	93	8.6
Well or moderate	603	56.4	Unknown	31	2.9
Poor	466	43.6	T stage		
Tumor size, cm			T1	821	76.8
<3	590	55.2	T2	248	23.3
≥3	479	44.8	N stage		
Tumor location			N0	895	83.7
Lower	595	55.7	N1	174	16.3
Middle	138	12.9	TNM stage		
Upper	207	19.4	IA	635	59.4
Multiple	129	12.0	IB	434	40.6
Haemoglobin, g/l (median, IQR)	135 (53–174)		AC		
Albumin, g/l (median, IQR)	40 (20–52)		No (surgery alone)	925	86.5
WBC, 10^9^/l (median, IQR)	6.0 (2.7–16.6)		Yes (surgery + AC)	144	13.5
Neutrophils, 10^9^/l (median, IQR)	3.5 (0.7–14.6)		Pretreatment hypoalbuminemia		
Platelets, 10^9^/l (median, IQR)	217 (80–523)		No	895	83.7
dNLR			Yes	174	16.3
<2	797	74.6			
≥2	272	25.4			

IQR = interquartile range; AC = adjuvant chemotherapy; dNLR = neutrophil count to (white cell count minus neutrophil count); ASA = American Society of Anesthesiologists. Pretreatment hypoalbuminemia: pretreatment serum albumin < 35 g/l.

**Table 2:** Patient baseline characteristics (adjuvant chemotherapy with surgery versus surgery alone).

Characteristics	AC group (*n* = 144)	Non-AC group (*n* = 925)	*p* value
Age, years (median, IQR)	58 (18–81)	60 (20–93)	0.402
Sex			0.474
Male	103	692	
Female	41	233	
CEA level			0.146
Normal	112	759	
Above normal	21	95	
Unknown	11	71	
CA 19–9 level			0.701
Normal	111	715	
Above normal	22	130	
Unknown	11	80	
Differentiation			
Well or moderate	78	525	
Poor	66	400	
Tumor size, cm			0.419
<3	84	506	
≥3	60	419	
Tumor location			0.591
Lower	76	519	
Middle	20	118	
Upper	31	176	
Multiple	17	112	
Type of surgery			0.522
Total gastrectomy	43	241	
Subtotal gastrectomy	101	684	
TNM stage			<0.001
IA	48	587	
IB	96	338	
Pretreatment dNLR			0.064
<2	116	681	
≥2	28	244	
Pretreatment hypoalbuminemia			0.260
No	121	774	
Yes	23	151	

**Table 3:** Univariate and multivariate Cox proportional analysis for survival.

Parameter	Categories	Univariate analysis		Multivariate analysis	
		HR (95% CI)	*p*	HR (95% CI)	*p*
Age	>65 versus <65	1.459 (0.974–2.186)	0.067		
Gender	Male versus female	1.408 (0.853–2.327)	0.181		
Tumor location	Upper versus other	1.254 (0.779–2.019)	0.351		
Tumor size	≥30 mm versus <30 mm	1.555 (1.041–2.324)	0.031	1.302 (0.731–1.816)	0.452
Lymphovascular invasion	Yes versus no	2.660 (1.509–4.689)	0.001	2.314 (1.419–4.417)	0.010
Tumor differentiation	Undifferentiated versus differentiated	1.392 (0.933–2.077)	0.105		
Extent of lymph node dissection	D1+/D1 versus D2	1.158 (0.643–1.391)	0.211		
AJCC TNM stage	IB versus IA	10.183 (5.765–17.987)	<0.001	8.098 (5.201–13.145)	<0.001
CEA level	Above normal versus normal	2.433 (1.440–4.110)	0.001	1.488 (0.873–2.426)	0.319
CA 19–9 level	Above normal versus normal	1.363 (0.789–2.356)	0.267		
Adjuvant chemotherapy	No versus yes	1.557 (0.784–3.092)	0.206		
dNLR	≥2 versus <2	4.357 (2.910–6.524)	<0.001	2.464 (1.301–4.312)	0.004
Pretreatment hypoalbuminemia	Yes versus no	2.181 (1.404–3.387)	0.001	2.102 (1.254–3.576)	0.028
SIS classification	2 versus ≤1	4.955 (3.175–7.733)	<0.001	3.023 (1.977–6.501)	0.002

CA: carbohydrate antigen; CEA: carcinoembryonic antigen; CI: confidence interval; HR: hazard ratio.

## Data Availability

The data used to support the findings of this study are available from the corresponding author upon request.

## Supplementary Materials

Supplementary 1 Table 1: characteristics of patients with stage I gastric cancer treated with gastrectomy for two treatment groups.

Supplementary 2 Figure 1: Kaplan-Meier curves for OS of stage I gastric cancer patients according to tumor stage.

Supplementary 3 Figure 2: the association between the SIS and TNM, depicted as a bubble chart.
