# Supplementary material for: Significance of Preoperative Systemic Immune Score for Stage I Gastric Cancer Patients
Source: Gastroenterol Res Pract. 2018 Jul 11;2018:3249436. doi: 10.1155/2018/3249436 (PMC6079442; doi:10.1155/2018/3249436)
Supplement: Supplementary 1 — Table 1: characteristics of patients with stage I gastric cancer treated with gastrectomy for two treatment groups. [file 3249436.f1.doc]

| Characteristic | Surgery alone | | Surgery plus AC | | p value |
| --- | --- | --- | --- | --- | --- |
|  | No. | % | No. | % |  |
| Age |  |  |  |  | 0.925 |
| ≥65 | 322 | 34.8 | 51 | 35.4 |  |
| ＜65 | 603 | 65.2 | 93 | 64.6 |  |
| Gender |  |  |  |  | 0.219 |
| Male | 694 | 75.0 | 101 | 70.1 |  |
| Female | 231 | 25.0 | 43 | 29.9 |  |
| Tumor location |  |  |  |  | 0.361 |
| Upper | 173 | 18.7 | 34 | 23.6 |  |
| Middle | 119 | 12.9 | 19 | 13.2 |  |
| Lower | 522 | 56.4 | 73 | 50.7 |  |
| Mixed | 111 | 12.0 | 18 | 12.5 |  |
| Tumor size |  |  |  |  | 0.857 |
| ＜3cm | 509 | 55.0 | 81 | 56.3 |  |
| ≥3cm | 416 | 45.0 | 63 | 43.8 |  |
| Lymphovascular invasion |  |  |  |  | 0.003 |
| Yes | 50 | 5.5 | 18 | 12.8 |  |
| No | 862 | 94.5 | 123 | 87.2 |  |
| Tumor differentiation |  |  |  |  | 1.000 |
| Differentiated | 522 | 56.4 | 81 | 56.3 |  |
| Undifferentiated | 403 | 43.6 | 63 | 43.8 |  |
| CEA level |  |  |  |  | 0.005 |
| Elevated | 99 | 10.7 | 28 | 19.4 |  |
| Normal | 826 | 89.3 | 116 | 80.6 |  |
| CA 19-9 level |  |  |  |  | 0.044 |
| Elevated | 130 | 14.1 | 30 | 20.8 |  |
| Normal | 795 | 85.9 | 114 | 79.2 |  |
| AJCC TNM stage |  |  |  |  | ＜0.001 |
| IA | 585 | 63.2 | 50 | 34.7 |  |
| IB | 340 | 36.8 | 94 | 65.3 |  |

Supplementary Table 1. Characteristics of patients with stage I gastric cancer treated with gastrectomy for two treatment groups

AC: adjuvant chemotherapy.
