# Supplementary figures and images for: Significance of Preoperative Systemic Immune Score for Stage I Gastric Cancer Patients
Source: Gastroenterol Res Pract. 2018 Jul 11;2018:3249436. doi: 10.1155/2018/3249436 (PMC6079442; doi:10.1155/2018/3249436)

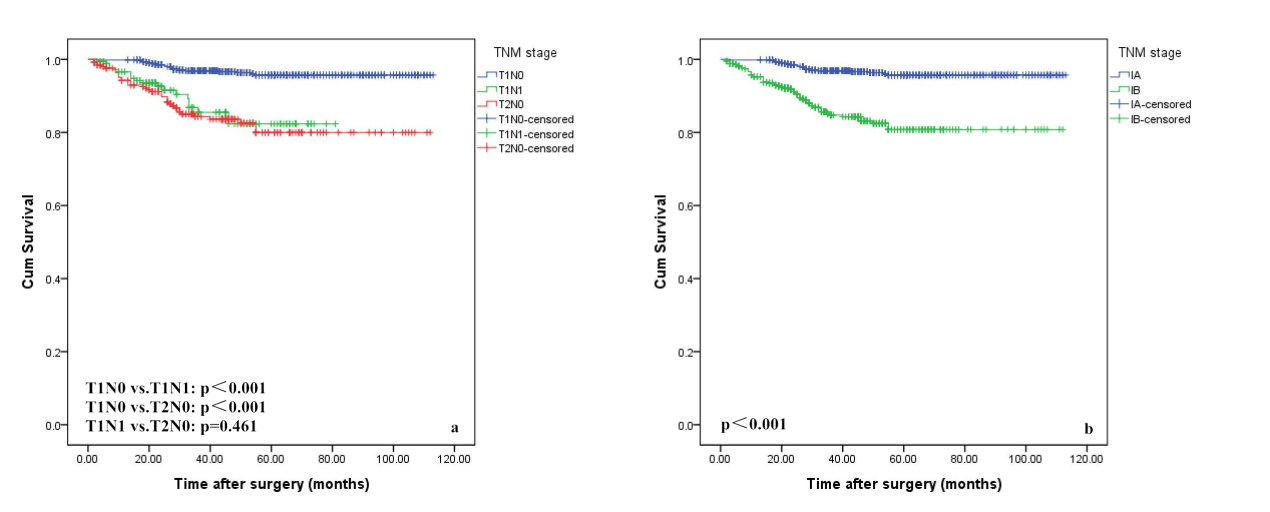

Supplement: Supplementary 2 — Figure 1: Kaplan-Meier curves for OS of stage I gastric cancer patients according to tumor stage. [file 3249436.f2.docx]

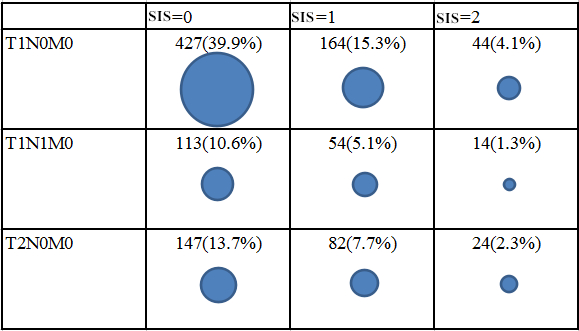

Supplement: Supplementary 3 — Figure 2: the association between the SIS and TNM, depicted as a bubble chart. [file 3249436.f3.docx]
